# The Association Between COVID-19 Mortality and ICU Admission Rates and Prior History of Angiotensin-Converting Enzyme Inhibitor or Angiotensin Receptor Blocker Use Among Hospitalized COVID-19 Patients With Hypertension in Michigan

**DOI:** 10.7759/cureus.43980

**Published:** 2023-08-23

**Authors:** Eiman Elhouderi, Eman Elsawalhy, Najlaa Al-Sudani, Irum Mahmood, David Sengstock

**Affiliations:** 1 Internal Medicine, Beaumont Health, Dearborn, USA; 2 Internal Medicine, Beaumont Hospital, Dearborn, USA

**Keywords:** mortality rate in covid-19, mortality and icu admission rates, icu admission, covid-19, hypertension, angiotensin receptor blockers, angiotensin-converting enzyme inhibitors

## Abstract

Importance

There are conflicting data regarding the safety of the use of angiotensin-converting enzyme inhibitor or angiotensin receptor blocker (ACEI/ARB) medications in hypertensive patients who are susceptible to COVID-19.

Objective

Our study assesses the association between COVID-19 severity and mortality and the use of ACEIs/ARBs among hospitalized patients with hypertension.

Research design, setting, and participants

This was a retrospective cohort study. Using the EPIC system of Beaumont Health, Dearborn, Michigan, we identified 5490 patients with COVID-19 who were admitted to the eight Beaumont hospitals. After excluding subjects who have no hypertension and those with missing data, we included 2129 COVID-19 patients who have hypertension. Logistic regression and Cox proportional hazards models were used to analyze the association between history of ACEI/ARB use, intensive care unit (ICU) admission rate, and COVID-19 mortality.

Exposure

Exposure refers to the use of ACEIs/ARBs as documented in the medical records before admission to the hospitals.

Main outcome

The main outcome was 30-day COVID-19 mortality and ICU admission rates.

Results

There were 1281 subjects (60%) with prior ACEI/ARB use and 848 subjects (40%) with no ACEI/ARB use. There was no significant association between ICU admission and the use of ACEIs/ARBs (odds ratio {OR} = 0.95, 95% CI = {0.76, 1.19}, p-value = 0.6). Although the unadjusted logistic regression model demonstrated a statistically significant association between history of ACEI/ARB use and COVID-19 mortality (odds ratio = 1.31, 95% CI = {1.05, 1.66}, p-value = 0.02), the adjusted logistic regression model failed to show this statistically significant association (odds ratio = 1.20, 95% CI = {0.93, 1.54}, p-value = 0.14). Moreover, we were not able to reveal a statistically significant association between 30-day COVID-19 survival and prior use of ACEI/ARB in the adjusted Cox proportional hazards model (hazard ratio {HR} = 1.11, 95% CI = {0.91, 1.40}, p-value = 0.14).

Conclusion

In this large retrospective study, we conclude that there was no statistically significant association between prior history of ACEI/ARB use and COVID-19 ICU admission rates or mortality in hypertensive patients hospitalized with COVID-19.

## Introduction

The number of people who got infected with the COVID-19 virus was around 43.7 million, and the number of deaths was around 710000 by October 2021 in the USA according to the Centers for Disease Control and Prevention (CDC) data tracker [1].

Recent data showed that patients with cardiovascular diseases such as hypertension who tested positive for COVID-19 had worse outcomes. Between 2017 and 2018, the prevalence of hypertension was 45% among adults in the USA. Millions of hypertensive patients use angiotensin-converting enzyme inhibitors (ACEIs) or angiotensin receptor blockers (ARBs) as the first-line treatment [2,3].

There are conflicting data regarding the use of these medications, the severity of the COVID-19 infection, and the mortality rate. The COVID-19 virus gains entry to pulmonary cells by binding to the membrane angiotensin-converting enzyme 2 (ACE-2) [4]. Hence, there is a concern about using ACEIs and ARBs as these medications use the same receptors. There is a debate regarding the increased risk of COVID-19 infection in patients who use ACEIs and ARBs by increasing the viral entry and subsequently increasing the viral load and the mortality risk. On the other hand, studies have shown that ACEI and ARB medications can protect against COVID-19 infection by attaching to the ACE receptors, thereby decreasing the availability of those receptors to the COVID-19 virus, leading to the deactivation of the virus and the decrease of the virus load [5-7]. Hence, many investigators have advised the use of ACEI and ARB medications in COVID-19-positive patients even with no history of hypertension. This study aims to further investigate the association between the use of ACEI/ARB prior to infection with the COVID-19 virus and COVID-19 mortality and severity.

This article was presented as a meeting abstract on May 26, 2021, at the 43rd annual research forum of the Southeast Michigan Center for Medical Education. This article was previously posted to the Authorea preprint server on January 30, 2023.

## Materials and methods

**Figure 1:**
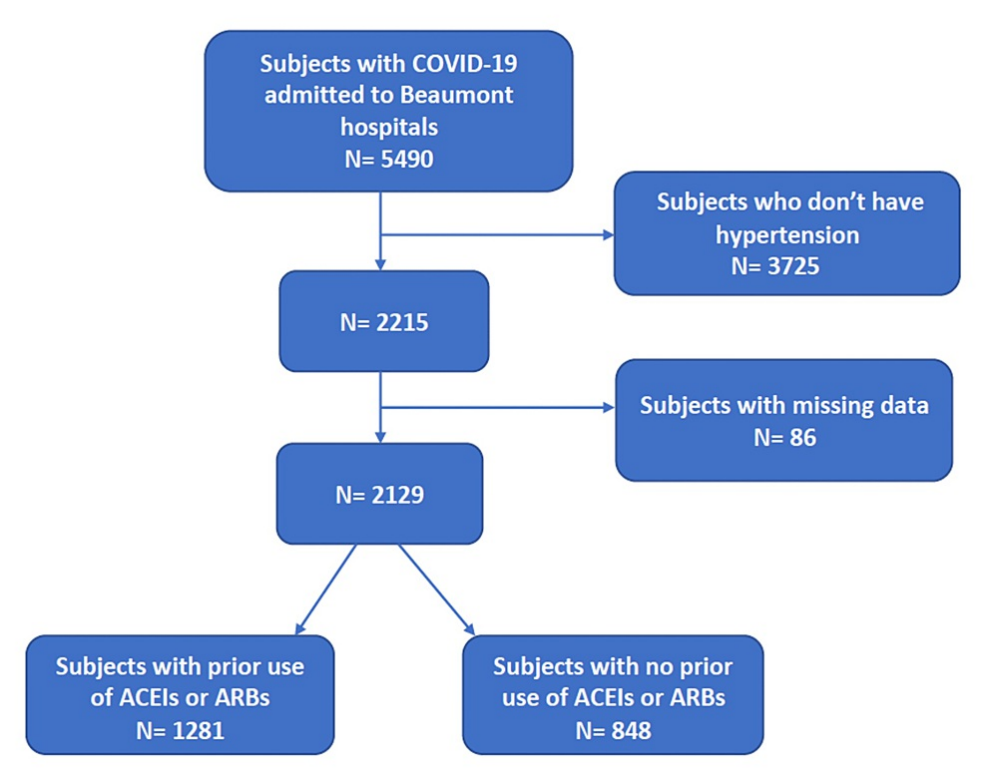
Patient flow chart ACEIs, angiotensin-converting enzyme inhibitors; ARBs, angiotensin receptor blockers

This was a retrospective cohort study. We researched the electronic health record system (EPIC system) of the Beaumont Health System, Dearborn, Michigan, for subjects who were admitted to the eight Beaumont hospitals and tested positive for COVID-19 from February 1, 2020, to April 30, 2020. We included only COVID-19 patients who have hypertension diagnoses in their charts. We excluded subjects with missing information on body mass index (BMI) (Figure 1). We retrieved information on the use of ACEIs and ARBs prior to admission to hospitals with COVID-19 diseases. We also obtained data on possible confounders including age, sex, race, BMI, diabetes mellitus (DM), chronic obstructive pulmonary disease (COPD), asthma, heart failure (HF), end-stage renal disease (ESRD), prior use of aldosterone antagonists, and the use of steroid during hospital admission. We followed subjects for 30 days after hospital admission. The two primary outcomes were 30-day COVID-19 mortality and intensive care unit (ICU) admission rates. We used the ICU admission rate as a marker of COVID-19 severity. We defined ICU admission as admission to intensive care units or progressive care units (PCU) since the admission criteria for both units include the use of invasive or noninvasive ventilatory support or vasopressors, which indicate severe disease.

Statistical analysis

We compared the baseline characteristics between subjects who had a history of ACEI/ARB use and those who did not use these medications using the chi-square test and t-test. We performed a simple logistic regression analysis to study the association between ICU admission rates and the use of ACEI/ARB. We then used a multivariate logistic regression model to adjust for possible confounders including age, sex, race, BMI, COPD, asthma, the use of aldosterone antagonists, heart failure, DM, and ESRD. Using the same methods, we studied the association between COVID-19 mortality and the use of ACEI/ARB. We used a Cox proportional hazards model to study the association between the use of ACEI/ARB and both 30-day COVID-19 survival and ICU admission rate. We censored the time to the event to 30 days. We first performed a univariate model and then performed a multivariate-adjusted model. We adjusted for the same possible confounders as those mentioned above.

R studio (Posit PBC, Boston, MA) was used to analyze the data, and a p-value of ≤0.05 was considered statistically significant.

## Results

We identified 5490 patients with COVID-19 who were admitted to the hospital. After excluding subjects with no hypertension and those with missing data, we included 2129 COVID-19 patients who had hypertension. Table 1 reveals the baseline subject characteristics of the two groups (a group that has a history of ACEI/ARB use and a group with no history of ACEI/ARB use). Both groups are similar in terms of sex, BMI, and the use of steroid during hospitalization. There were more patients with DM, heart failure, ESRD, asthma, and COPD in the ACEI/ARB group than in the non-ACE/ARB group.

**Table 1:** Baseline patients characteristics We used N (%) for categorical variables and mean (SD) for continuous variables HF, heart failure; DM, diabetes mellitus; BMI, body mass index; COPD, chronic obstructive pulmonary disease; ESRD, end-stage renal disease; ACEI, angiotensin-converting enzyme inhibitor; ARB, angiotensin receptor blocker

Variables	ACEI or ARB (N = 1281, 60%)	No ACEI or ARB (N = 848, 40%)	P-value
Age	68 (14)	66 (15)	0.007
Sex (female)	649 (50%)	442 (52%)	0.5
BMI	32.1 (8.3)	31.8 (8.8)	0.4
Race	African American = 680 (53%)	African American = 525 (61%)	0.0001
Asthma	176 (14%)	91 (10%)	0.04
COPD	125 (10%)	49 (6%)	0.001
HF	186 (16%)	83 (10%)	0.001
DM	628 (49%)	285 (33%)	<0.001
ESRD	76 (5%)	33 (4%)	0.03
Steroid use	627 (49%)	438 (52%)	0.2
Aldosterone antagonist	128 (10%)	23 (3%)	<0.01

A total of 532 (25%) patients were admitted to the ICU or PCU (319 subjects {25%} from the ACEI/ARB group and 213 {25%} subjects from the non-ACEI/ARB group). There was no statistically significant difference in ICU admission rates between the ACEI/ARB and non-ACEI/ARB groups in both adjusted (odds ratio {OR} = 1.02, 95% CI = {0.04, 0.22}, p-value = 0.7) and unadjusted models (odds ratio = 0.95, 95% CI = {0.76, 1.19}, p-value = 0.6) (Table 2).

**Table 2:** Adjusted logistic regression model exploring the association between the use of ACEIs/ARBs and ICU admission rates Adjusted for age, sex, body mass index (BMI), race, asthma, chronic obstructive pulmonary disease (COPD), heart failure (HF), diabetes mellitus (DM), end-stage renal disease (ESRD), aldosterone antagonist, and steroid use ACEI, angiotensin-converting enzyme inhibitor; ARB, angiotensin receptor blocker; ICU, intensive care unit

Variable	Odds ratio for ICU admission rate	95% CI	P-value
ACEI/ARB user (non-ACEI/ARB user as a reference group)	1.02	(0.04, 0.22)	0.7
Age	1.01	(1.002, 1.02)	0.01
Sex (female)	0.89	(0.72, 1.08)	0.23
BMI	0.99	(0.98, 1.01)	0.4
Race (African American)	1.32	(1.06, 1.64)	0.01
Asthma	0.67	(0.48, 0.92)	0.01
COPD	1.08	(0.75, 1.54)	0.6
HF	1.22	(0.90, 1.66)	0.2
DM	1.43	(1.18, 1.75)	<0.01
ESRD	2.45	(1.62, 3.80)	<0.01
Steroid use	3.45	(2.83, 4.24)	<0.01
Aldosterone antagonist	1.02	(0.69, 1.49)	0.9

A total of 391 patients (18%) passed away within 30 days of admission (256 {20%} subjects from the ACEI/ARB group and 135 {16%} subjects from the non-ACEI/ARB group). Although there was a statistically significant difference in the COVID-19 mortality rates between the two groups in the unadjusted logistic regression model (odds ratio = 1.31, 95% CI = {1.01, 1.54}, p-value = 0.03), the difference in the odds ratio of COVID-19 mortality between the two groups was not statistically significant in the adjusted model (odds ratio = 1.20, 95% CI = {0.93, 1.54}, p-value = 0.14) (Table 3).

**Table 3:** Adjusted logistic regression model exploring the association between the use of ACEIs/ARBs and COVID-19 mortality Adjusted for age, sex, body mass index (BMI), race, asthma, chronic obstructive pulmonary disease (COPD), heart failure (HF), diabetes mellitus (DM), end-stage renal disease (ESRD), aldosterone antagonist, and steroid use ACEI, angiotensin-converting enzyme inhibitor; ARB, angiotensin receptor blocker

Variable	Odds ratio for COVID-19 mortality	95% CI	P-value
ACEI/ARB user (non-ACEI/ARB user as a reference group)	1.20	(0.93, 1.54)	0.14
Age	1.06	(1.05, 1.07)	<0.01
Sex (female)	0.85	(0.65, 1.07)	0.16
BMI	1.01	(0.98, 1.023)	0.47
Race (African American)	0.84	(0.66, 1.1)	0.23
Asthma	0.93	(0.62, 1.37)	0.74
COPD	1.07	(0.72, 1.57)	0.72
HF	1.19	(0.85, 1.66)	0.28
DM	1.30	(1.02, 1.66)	0.03
ESRD	1.30	(0.75, 2.17)	0.32
Steroid use	2.82	(2.20, 3.64)	<0.01
Aldosterone antagonist	1.03	(0.64, 1.60)	0.9

**Figure 2:**
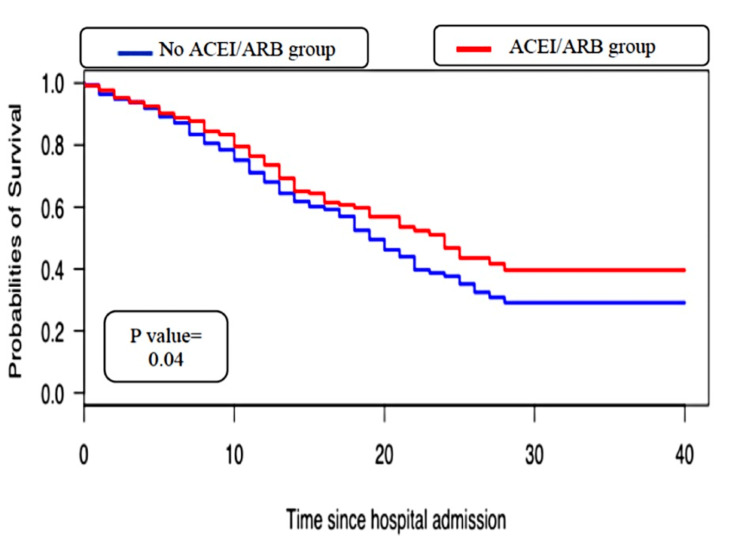
Kaplan-Meier curve demonstrating the association between the use of ACEI/ARB and COVID-19 mortality ACEI, angiotensin-converting enzyme inhibitor; ARB, angiotensin receptor blocker

The Kaplan-Meier curve illustrated that ACEIs/ARBs were associated with increased COVID-19 mortality (Figure 2). Similarly, the unadjusted Cox proportional hazards model showed that the use of ACEI/ARB was associated with an increase in COVID-19 mortality (hazard ratio {HR} = 1.25, 95% CI = {1.01, 1.54}, p-value = 0.03). However, we failed to find a statistically significant association between the history of ACEI/ARB use and 30-day COVID-19 survival in the adjusted Cox proportional hazards model (Table 4).

**Table 4:** Adjusted proportional hazards model illustrating the association between the use of ACEI/ARB and COVID-19 mortality Adjusted for age, sex, body mass index (BMI), race, asthma, chronic obstructive pulmonary disease (COPD), heart failure (HF), diabetes mellitus (DM), end-stage renal disease (ESRD), aldosterone antagonist, and steroid use ACEI, angiotensin-converting enzyme inhibitor; ARB, angiotensin receptor blocker

Variable	Hazard ratio for COVID-19 mortality	95% CI	P-value
ACEI/ARB user (non-ACEI/ARB user as a reference group)	1.12	(0.91, 1.40)	0.14
Age	1.04	(1.037, 1.05)	<0.01
Sex (female)	0.90	(0.73, 1.11)	0.34
BMI	0.99	(0.98, 1.01)	0.72
Race (African American)	0.83	(0.67, 1.03)	0.83
Asthma	1.01	(0.72, 1.40)	0.97
COPD	1.20	(0.87, 1.66)	0.24
HF	0.95	(0.72, 1.25)	0.73
DM	1.15	(0.94, 1.41)	0.16
ESRD	0.87	(0.55, 1.37)	0.56
Steroid use	1.01	(0.83, 1.31)	0.69
Aldosterone antagonist use	1.09	(0.75, 1.58)	0.63

The Cox proportional hazards model showed no significant association between ACEI/ARB use and the time to ICU admission in both the unadjusted (HR = 0.9, 95% CI = {0.8, 1.1}, p-value = 0.5) and adjusted (HR = 0.9, 95% CI = {0.79, 1.11}, p-value = 0.5) models (Table 5).

**Table 5:** Adjusted proportional hazards model illustrating the association between the use of ACEI/ARB and ICU admission rates Adjusted for age, sex, body mass index (BMI), race, asthma, chronic obstructive pulmonary disease (COPD), heart failure (HF), diabetes mellitus (DM), end-stage renal disease (ESRD), aldosterone antagonist, and steroid use ACEI, angiotensin-converting enzyme inhibitor; ARB, angiotensin receptor blocker; ICU, intensive care unit

Variable	Hazard ratio for ICU admission rate	95% CI	P-value
ACEI/ARB user (non-ACEI/ARB user as a reference group)	0.9	(0.79, 1.11)	0.5
Age	1.01	(0.99, 1.01)	0.1
Sex (female)	1.06	(0.90, 1.25)	0.4
BMI	0.99	(0.98, 1.00)	0.2
Race (African American)	1.02	(0.86, 1.22)	0.7
Asthma	0.8	(0.67, 1.16)	0.4
COPD	1.07	(0.80, 1.43)	0.6
HF	1.01	(0.79, 1.28)	0.9
DM	0.98	(0.83, 1.16)	0.8
ESRD	1.09	(0.80, 1.49)	0.5
Steroid use	0.65	(0.55, 0.78)	<0.01
Aldosterone antagonist	0.9	(0.73, 1.35)	0.9

## Discussion

Among patients who were hospitalized for COVID-19, this study showed no significant association between prior use of ACEIs/ARBs and COVID-19 mortality or severity as evidenced by ICU admission rate after adjusting for baseline characteristics, comorbidities, aldosterone antagonist use, and steroid use, using the adjusted logistic regression model and Cox proportional hazards model.

The mean BMI in our study population was high (32). Eighty percent of the patients had a BMI equal to or higher than 25. This finding correlates with the findings of other studies on the association between BMI and COVID-19 hospitalization rates [8].

A retrospective cohort study was conducted in two Saudi public specialty hospitals and included 354 patients with COVID-19 (146 used ACEI/ARB in the last six months prior to COVID-19 infection, and 208 were non-ACEI/ARB users). This study showed that the ACEI/ARB group had an eightfold higher risk of developing critical or severe COVID-19 (OR = 8.25; 95% CI = {3.32, 20.53}), nearly sevenfold higher risk of intensive care unit (ICU) admission (OR = 6.76; 95% CI = {2.88, 15.89}), and a nearly fivefold higher risk of requiring noninvasive ventilation (OR = 4.77; 95% CI = {2.15, 10.55}). However, this study had a small sample size, and it overlooked some confounders including the use of steroids [9].

On the other hand, a prospective cohort study in England involving 8.28 million participants showed that exposure to ACEI/ARB in the last 90 days is associated with reduced risk of COVID-19-positive disease requiring hospitalization. This study is limited by the unavailability of a standard systematic strategy for COVID-19 testing in the United Kingdom and restricting the COVID-19 testing to only hospitalized patients by UK health policy during the period of the study at that time [10].

Another study conducted in New York in March 2020 showed a reduced length of hospital stay in patients admitted with COVID-19 who used ACEI/ARB in the hospital, most of whom belonged to ethnic minorities. The limitations of this study include the disproportionate size of the control versus the treatment group and the fact that the study only considered the ACEI/ARB during hospitalization [11].

Moreover, a meta-analysis including 13 studies conducted in 2020 showed that ACEI/ARB use was not associated with an increased risk of all-cause mortality or severe disease in patients with COVID-19 infection. Among these 13 studies, 11 studies showed that the ACEI/ARB group had a significantly lower risk of all-cause mortality than the non-ACEI/ARB group. This meta-analysis was limited by inconsistencies in the definition of severe diseases and in inclusion criteria [12].

In addition to this meta-analysis, an open randomized controlled trial in Brazil including 659 hospitalized patients with mild to moderate COVID-19 showed no effect of the discontinuation of ACEIs or ARBs on COVID-19 mortality [12]. However, this study explored a different question than our study. The two proposed conflicting hypotheses state that either ARBs and ACEIs increase ACE-2 expression, allowing virus entry into the cell and, therefore, increasing SARS-CoV-2 infectivity and severity, or coronavirus downregulates ACE-2 receptors causing an elevation of angiotensin II, which may lead to lung injury. Based on this, prior use of ACEI/ARB would affect COVID-19 infectivity, severity, or mortality than the use of ACEIs/ARBs after the establishment of COVID-19 [13].

We have a large high-quality database of hospitalized COVID-19 patients at our hospitals. Our study is observational, and we were able to reduce the confounding effect by adjusting a wide range of confounders recorded in electronic medical records including age, race, BMI, and other comorbidities. Our sample was representative and was restricted to a group of patients with hypertension. Another great strength of our study is the inclusion of a diverse group of patients as the eight hospitals are spread out over the Southeast Michigan region where a majority of Michigan’s population resides and comprises Caucasians, African Americans, and Arabs. The limitations of our study include the retrospective nature of the study, the inability to specify the chronicity of ACEI/ARB use based on the available data, overlooking of unknown confounders, and, finally, performing the study during the early months of the COVID-19 pandemic where little information about the treatment and disease was available.

## Conclusions

In this large retrospective study, we found that there is no association between exposure to ACEI/ARB prior to COVID-19 infection and 30-day COVID-19 mortality among hypertensive patients admitted with COVID-19 to the eight Beaumont hospitals in Michigan after controlling for demographic factors, medical comorbidities, aldosterone antagonist use, and steroid use. Our analysis also showed that prior exposure to ACEI/ARB was not associated with COVID-19 ICU admission rate. Therefore, we conclude that there is no association between prior history of ACEI/ARB use and COVID-19 severity or mortality. This study together with prior literature provides strong supportive evidence for the safe use of ACEI/ARB in the treatment of hypertension.
